# Mind the Gap!

**DOI:** 10.1371/journal.pbio.1000122

**Published:** 2009-06-02

**Authors:** Antonio Lazcano

**Affiliations:** Facultad de Ciencias, Universidad Nacional Autónoma de México (UNAM), Mexico City, Mexico

## Abstract

In his review of *Protocells*, Antonio Lazcano explores how the self-assemblage properties of lipids into bilayers, micelles, and liposomes can be used to model minimal cells, design biochemical microreactors, and provide insights into the predecessors of the first living beings.

In 1835, the French naturalist Felix Dujardin started crushing ciliates under the microscope and observed that the tiny cells exuded a jellylike, water-insoluble substance, which he described as a “*gelée vivante*” and which was eventually christened “protoplasm” by the physician Johann E. Purkinje and the botanist Hugo von Mohl. Fifty years after Dujardin's observations, the possibility that living organisms were the evolutionary outcome of the gradual transformation of lifeless gel-like matter into protoplasm was so widespread that it found its way into musical comedies. In 1885, the self-important Pooh-Bah, Lord Chief Justice and Chancellor of the Exchequer, declared in Gilbert and Sullivan's *The Mikado* that “I am in point of fact, a particularly haughty and exclusive person, of pre-Adamite ancestral descent. You will understand this when I tell you that I can trace my ancestry back to a protoplasmal primordial atomic globule.”

As a number of contributors argue in the book *Protocells: Bridging the nonliving and living matter* (edited by Steen Rasmussen, Mark A. Bedau, Liaohai Chen, David Deamer, Norman H. Packard, and Peter F. Stadler), life on Earth may have descended from primordial globules formed in the primitive oceans. This thick volume collects papers by 31 authors that were presented at two different international workshops, in which theoretical and experimental models of protocells were analyzed in considerable detail. As summarized by molecular biologist Martin M. Hanczyc in the opening chapter, mesmerizing reports of lifelike behaviors by microscopic droplets of different compositions led Jerome Alexander, Stéphane Leduc, and Alfonso L. Herrera to the conclusion that physicochemical models of protoplasm might provide insights into the origin of life. Other scientists, like the Dutch biochemist Bungenberg de Jong, argued that the colloid properties of droplets, which he termed coacervates, formed by the spontaneous aggregation of biological macromolecules, could explain the properties of protoplasm. And in a book published in Russian in 1936, Aleksandr I. Oparin went further and proposed these droplets as precursors of the first cells. Oparin considered enzymatic-based assimilation, growth, and reproduction traits of life, but not nucleic acids, whose role as the material basis of inheritance was not even suspected. He assumed that biological inheritance was the outcome of growth and division of the coacervate drops that he viewed as models of precellular systems. In hindsight, this may seem naïve, but Oparin's commitment to coacervates resulted in part from his refusal to assume that life can be reduced to a single compound such as the randomly formed “living gene” proposed by the American geneticist Herman Muller.

Thirty years ago, laboratory simulations and the discovery of a large array of amino acids and nucleobases in meteorites had reinforced the idea that life arose from a primitive soup, but few dared to discuss how the huge gap separating organic molecules of prebiotic origin from the first living systems could be closed. Oparin's coacervates were seen by many as historical curiosities with little, if any, significance for the study of the appearance of life, which had become focused on the origin of genetic replication. Whether the earliest genetic polymers were enclosed within membranes is not yet clear, but as summarized by biochemist David W. Deamer in this volume, he and others, like the late Spanish chemist Juan Oró, were convinced that this was the case. Their assumptions were buttressed over 30 years ago when they independently achieved the abiotic synthesis of lipids under mild conditions; they were reinforced a few years later when Deamer and Pashley reported the presence of membrane-forming lipidic compounds in a sample of the Murchison meteorite. Lipids slowly but steadily began to glide toward origin-of-life scenarios and, at the same time, started to receive attention from industry, as when it was shown that micelles and liposomes behave as microreactors and could be used to deliver interferon and other medicaments into cells.

There are more things in lipids that are dreamt of in our laboratories. The spontaneous self-assembly of amphiphilic molecules into bilayers, micelles, and vesicles are well-known, but as the ETH-based chemist Pier Luigi Luisi and his associates write in this book, 20 years ago he and his colleagues demonstrated the feasibility of autocatalytic formation and self-replication of micelles. Micelles and liposomes do not reproduce and do not store genetic information, but they replicate by a mechanism that differs from that of nucleic acids, raising new questions in our understanding of the origin of cells.

As the editors of *Protocells* state in the Introduction, “in contrast to astrobiology, protocell research does not need to justify the cosmochemical or geochemical origins of its starting materials.” This is true, but the papers that address the origin of life and cell precursors are among the most appealing ones in this book, describing the different experimental efforts to obtain systems capable of both efficient catalysis and self-replication. As Frazier and his colleagues argue in their chapter on possible applications of cell-like entities, there are several attempts to “create novel technologies for civilian and military applications, including smart biosensors that are able to respond to changes in the environment, tracking devices to locate personnel, and monitoring devices to detect physiological change with built-in response systems to modulate any adverse conditions that may be detected.” These are great expectations, but as summarized in several chapters, this is a promising field which already intersects with the study of minimal life, chemical dynamics, self-organizing phenomena, and potential practical applications, many of which fall within the rapidly expanding limits of what is now called synthetic biology. This book closes with a rather odd chapter by Mark A. Bedau and Emily C. Parke, both of the biotech company ProtoLife SRL, on the social and ethical implications of protocell research, which appears to be written for the sake of philosophical correctness. Like in many other areas of research related to life sciences, future developments in protocell and artificial cell investigations may raise questions for which our current social, legal, and ethical frameworks do not provide straightforward answers—but recognizing the essential differences between ethical and moral (i.e., religiously based) concerns would probably have a larger appeal to a wider, more cosmopolitan scientific audience.

This volume has been carefully produced and well edited, and includes a rather useful glossary, but unfortunately it took more than 2 years to get published. For example, while this book was in preparation, the experimental system that could combine vesicle replication with nonenzymatic nucleic acid polymerization proposed by the Harvard-based team lead by Jack Szostak that is included in this volume led to a major 2008 publication showing that such a laboratory model of protocell-like systems was feasible, adding more weight to the role of lipids and membranes in the emergence of cells.

We do not know how life appeared on Earth, or if precellular systems like liposomes played a role in it. The reader should not feel overwhelmed with the sheer scale of the questions to be solved but, as shown by the contributions that form this volume, encouraged by the ingenuity with which those issues can be confronted. As the late Leslie Orgel once wrote, things that stay together tend to evolve together. The different contributors to this volume advocate this idea, but at times one feels that the editors provide too much of a good thing: lipids were probably present in the primitive environment and are excellent for developing models of cell-like entities, but it's a pity that other possible compartments have not been sufficiently discussed. This book raises many questions that are left unanswered, but one can hope that it will lead to more discussions and research in years to come.[Fig pbio-1000122-g001]

**Figure pbio-1000122-g001:**
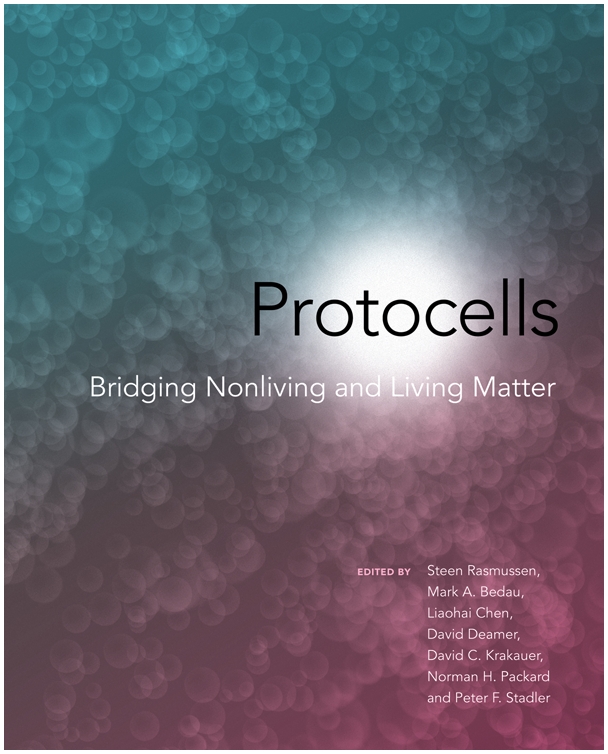
Rasmussen S, Bedau MA, Chen L, Deamer D, Packard NH, et al., editors (2008) Protocells: Bridging nonliving and living matter. Cambridge (Massachusetts): MIT Press. 776 p. ISBN (hardcover): 978-0262182683. US$75.00.

